# Antifungal Activity of Essential Oil Compounds (Geraniol and Citral) and Inhibitory Mechanisms on Grain Pathogens (*Aspergillus flavus* and *Aspergillus ochraceus*)

**DOI:** 10.3390/molecules23092108

**Published:** 2018-08-22

**Authors:** Xi Tang, Ye-Lin Shao, Ya-Jie Tang, Wen-Wen Zhou

**Affiliations:** 1 College of Biosystems Engineering and Food Science, Fuli Institute of Food Science, Zhejiang Key Laboratory for Agro-Food Processing, Key Laboratory for Agro-Products Postharvest Handling of Ministry of Agriculture, Zhejiang University, Hangzhou 310058, China; 21613043@zju.edu.cn (X.T.); shzoyelin@126.com (Y.-L.S.); 2 Key Laboratory of Fermentation Engineering (Ministry of Education), Hubei Provincial Cooperative Innovation Center of Industrial Fermentation, Hubei Key Laboratory of Industrial Microbiology, Hubei University of Technology, Wuhan 430068, China; yajietang@hotmail.com

**Keywords:** antifungal activity, geraniol, citral, *Aspergillus*, relative electric conductivity, reactive oxygen species, gene expression, modified atmosphere packaging

## Abstract

The grain contamination by *Aspergillus* spp. has been a serious issue. This study exhibited the excellent antifungal effects of the essential oil compounds (EOCs) geraniol and citral against common grain pathogens (*A. flavus* and *A. ochraceus*) in vitro and in situ. The inhibitory mechanisms were also evaluated from the perspective of cell membrane permeability, reactive oxygen species (ROS) generation, and *Aspergillus* spp. growth-related gene expression. Meanwhile, the combined effects of EOCs in the vapor phase and modified atmosphere packaging (MAP) were examined to find an alternative preservation method for controlling *Aspergillus* spp. The results indicated that citral exhibited the antifungal activity mainly by downregulating the sporulation- and growth-related genes for both pathogens. Geraniol displayed inhibitory effectiveness against *A. flavus* predominantly by inducing the intracellular ROS accumulation and showed toxicity against *A. ochraceus* principally by changing cell membrane permeability. Furthermore, the synthetic effects of EOCs and MAP (75% CO_2_ and 25% N_2_) induced better grain quality than the current commercial fumigant AlP. These findings reveal that EOCs have potential to be a novel grain preservative for further application.

## 1. Introduction

Grain is a precious resource for mankind’s survival, and it is also a country’s economic lifeline. However, during the storage period, food may become corrupted and inedible, resulting in substantial economic losses and threatening human life. The causes of food deterioration include pests and molds, especially in developing countries. Fungal contamination of stored seeds and grains is a serious chronic problem in the Indian storage system due to the tropical and humid climate [1]. Harvested grains are prone to infestations of different types of *Aspergillus* spp., resulting in spoilage and mycotoxin contamination under particular conditions. *Aspergillus* spp. are the most typical cereal spoilage fungal species and can produce some toxic secondary metabolites such as mycotoxins in food and feedstuff. Fungal growth and relevant mycotoxin production may lead to food spoilage and affect the quality and quantity of food to various degrees [2].

In developing countries, cereal preservation is generally achieved by using chemical preservatives and controlling the storage environment. The preservatives currently used in the food industry are mostly chemically synthesized organic acids and relevant salts as well as some antioxidants. For example, methyl bromide and phosphine used in chemical fumigation measures are commonly applied worldwide to control pest and mold infestations in warehouses. However, this method has a negative impact for the environment and is a threat to human health. Thus, there is an urgent need to find a practical, effective, and nonpolluting method to replace traditional chemical fumigants to control fungal contamination of stored grain.

Natural products such as essential oils (EOs), flavonoids, alkaloids, and polysaccharides have attracted numerous researchers’ attention owing to their antimicrobial, antitumor, and anti-inflammatory functions. EOs are complex mixtures of volatile compounds extracted from plants, and the main components of EOs include aromatic and aliphatic constituents which are characterized by low molecular weight [3]. They are biosynthesized by all plant organs, such as flowers, leaves, stems, seeds, fruits, roots, wood, bark, and so forth; are usually distributed in secretory cells, cavities, canals, and epidermic cells; and can be obtained by distillation and pressing. Regarding mode of action for cells, because of a vast number of complex compound constituents, EOs appear to have no specific cellular targets [4]. Owing to the complexity of the inhibitory action of EOs, it is time that the EO compounds (EOCs) should be taken into consideration to clarify the antimicrobial mechanism in detail. As typical lipophilic compounds, some related EOCs could pass through the cell wall and lipid-based cytoplasmic membrane, disturbing the structure of multilayered polysaccharides, fatty acids, and phospholipids, thereby destroying the overall cellular structure. Membrane damage is considered to be a type of cytotoxicity characteristic of EOCs. In eukaryotic cells, cell membranes and mitochondrial membranes control the ingress and egress of different components, such as ionic Ca^2+^ cycling [5] and other ionic channels. EOCs might affect the membrane expansion and change the membrane fluidity by interfering with the proton pump and the ATP pool to cause the disordering of intracellular macromolecular substances (such as nucleic acids and proteins), intracellular enzyme activities, and energy metabolism. This mode of action of EOCs might be directed to a single target or multiple targets in the cell. Once this interaction mode is triggered, anabolism is promoted or catabolism is inhibited in the cell, resulting in an imbalance between synthesis and catabolism, which in turn leads to the accumulation of energy substances. Moreover, the intracellular respiratory rate, phosphorylation, and oxidative phosphorylation efficiency are also inhibited, leading to cell death by apoptosis and necrosis [6].

At present, some countries have used atmospheric storage technologies to store food crops. Molds are the main corrupting organisms in the grain storage ecosystem and are essentially aerobic. Consequently, creating an oxygen-deficient environment in the storage grain ecosystem has a fatal effect on molds and greatly extends the shelf life. The use of a modified atmosphere to store food crops involves changing the natural stored gas composition, which includes carbon dioxide (CO_2_), oxygen (O_2_), and nitrogen (N_2_), and is lethal to pathogens. In the literature, common atmospheric storage technologies can be divided into modified atmosphere (MA) and controlled atmosphere (CA) categories [7]. Their difference is the degree of atmospheric composition. The gas composition of the artificially controlled storage is initially adjusted and dynamically changes according to the respiration rate of the crops and the permeability of the membrane or storage structure around the grain. The gas composition has been constantly controlled and monitored during the storage period. In addition to extending the storage period, atmospheric storage technology can also maintain the natural quality of the grain. Changing the gas around the food can reduce the respiration rate of the grain and inhibit the activity of microorganisms in the grain, thereby greatly prolonging the storage life of the grain.

However, there is very limited research that has studied the mode of interaction of EOCs and *Aspergillus* spp. from the gene level and the function of EOCs as the grain preservative. Thence, the objective of this work was to determine the efficacy of diverse EOCs against *A. flavus* and *A. ochraceus* and to reveal the underlying mechanism of antimicrobial activities from the aspects of cell membrane morphology, intracellular reactive oxygen species (ROS) change, and differential gene expression. Moreover, EOCs were investigated in combination with modified atmosphere packaging (MAP) to further improve the grain quality regarding *Aspergillus* infection.

## 2. Results

### 2.1. Inhibitory Effects of Geraniol and Citral on Toxigenic Fungi

Among all the tested EOs’ major components, there were several vapors of EOCs with effective inhibitory activity against *A. flavus* and *A. ochraceus* in vitro (Supplementary Table S1). Unfortunately, the other components showed little antifungal property for the target pathogens. A possible explanation for this is that some EOCs have a special mode of action for some specific microbes. Geraniol and citral significantly inhibited mycelial growth (*p* < 0.05) and exhibited higher inhibition rates for two strains compared to other treatments. Table 1 presents the antifungal effect with different concentrations of geraniol and citral at the seventh day after inoculation with spoilage pathogens. The minimum inhibitory concentration (MIC) of citral was 0.5 μL/mL for *A. flavus* and 0.4 μL/mL for *A. ochraceus*, whereas geraniol seemed not to fully prevent the growth of these fungi. The inhibition rate of geraniol against *A. flavus* was 98.44% between 0.5 and 0.6 μL/mL, and similarly, 98.38% against *A. ochraceus* at the range of 0.3 to 0.6 μL/mL. It was indicated that geraniol in the vapor phase might reach saturated concentration in sealed plates. Considering excellent in vitro results, geraniol and citral were chosen for subsequent in situ studies.

### 2.2. Influence of EOC Treatments on Grain Quality Parameters

**Figure 1:**
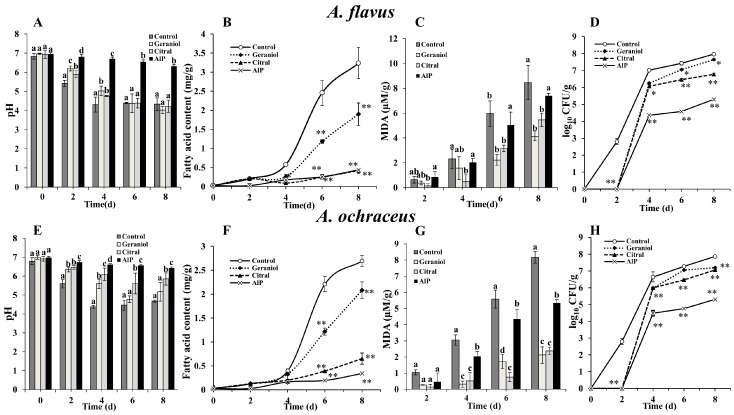
Effects of geraniol and citral on physiological qualities of rice infected by *A. flavus* and *A. ochraceus*. The grains were stored for various time intervals (0, 2, 4, 6, and 8 d) with geraniol (0.5 μL/mL), citral (0.5 μL/mL), and AlP (10 mg/L) treatment and sterile water (control), respectively. (**A**) Changes in pH values with *A. flavus*; (**B**) Changes in fatty acid content with *A. flavus*; (**C**) Changes in MDA content with *A. flavus*; (**D**) Changes in colony number of *A. flavus*; (**E**) Changes in pH values with *A. ochraceus*; (**F**) Changes in fatty acid content with *A. ochraceus*; (**G**) Changes in MDA content with *A. ochraceus*; and (**H**) Changes in colony number of *A. ochraceus*. Data are the mean ± SD of three independent experiments. Different letters at each time point denote significant difference (*p* < 0.05), and an asterisk (*p* < 0.05) and two asterisks (*p* < 0.01) are used when compared with control according to Duncan’s test.

The pH with geraniol fumigation falls fast from 6.97 ± 0.03 to 4.02 ± 0.17 in eight days; a similar result was obtained with citral (Figure 1A). This was likely due to the two EOs’ major components in the vapor phase being slightly acidic. However, aluminium phosphide (AlP), a common commercial fumigant, could prominently maintain stable pH at the level of 6.54 ± 0.04 (*p* < 0.05). Fat acidity of grain treated with geraniol and citral was at the values of 1.89 ± 0.29 and 0.42 ± 0.07 NaOH mg/g, respectively, when compared to grain without EOCs after being infected with *A. flavus* for eight days (*p* < 0.01) (Figure 1B). Additionally, citral, equally to AlP, prevented the rapid rise of lipid acids of grains for both *Aspergillus* infections. There was a slowly increasing tendency of malondialdehyde (MDA) content in grains with EOC treatments and the content in the control group inoculated with *A. ochraceus* was 3.85 times that of citral at the eighth day (*p* < 0.05). It seemed that geraniol and citral, which were the major components of *Lippia rugosa* EO and lemongrass EO respectively, could better delay MDA increase of grain after inoculation with *A. ochraceus* than *A. flavus* (Figure 1C–G). Meanwhile, there was no substantial difference in MDA content between the AlP and control after eight days’ infections of *A. flavus*. For microbial numbers, geraniol and citral could significantly reduce colonization by *A. flavus* and *A. ochraceus* in grain after inoculation (*p* < 0.05) (Figure 1D–H). These results signified that geraniol and citral have the potential to protect grain (rice) from infection by pathogens and to delay the grains’ corruption.

### 2.3. Scanning Electron Microscopy (SEM) Analysis

**Figure 2:**
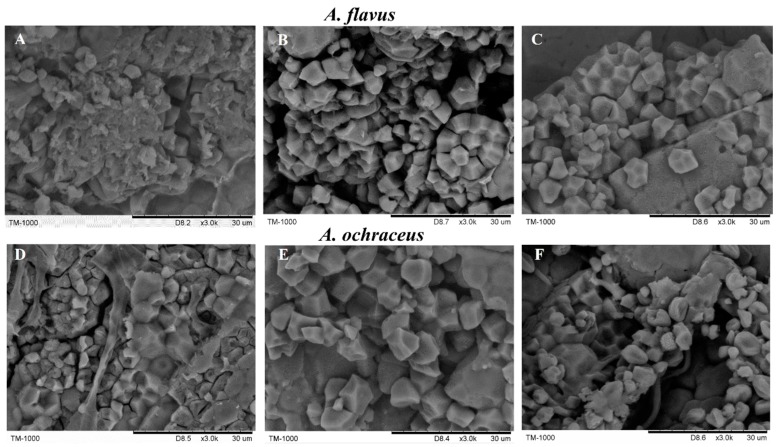
The SEM images of surface morphologies of rice with geraniol and citral treatments after 8 days of exposure to *Aspergillus* spp. (**A**) *A. flavus*; (**B**) *A. flavus* treated with geraniol; (**C**) *A. flavus* treated with citral; (**D**) *A. ochraceus*; (**E**) *A. ochraceus* treated with geraniol; and (**F**) *A. ochraceus* treated with citral.

The microstructure of interactions between starch granules and fungi were observed using SEM. SEM micrographs of *A. flavus* or *A. ochraceus* on grains after treatment with EOCs are shown in Figure 2. Many *A. flavus* mycelium and visible spores were distributed on the surface of granules, and the release of internal starch contents from boundary damage in grains was noticeable (Figure 2A). Similarly, *A. ochraceus* affected the integrity of starch granules seriously and resulted in some partial disruption of starches (Figure 2D). It was speculated that the mold occupied space gaps among starch granules and consumed nutrients, thus eroding the rice grains. After treatment with geraniol, starches appeared to follow healthy development with a regular crystalline form (Figure 2B) and were much larger than that in Figure 2A, revealing that geraniol not only effectively prevented the infection of *A. flavus* and *A. ochraceus* on grains, but also maintained the complete structure of granules without harmful side effects. As shown in Figure 2C–F, no apparent variation of the starch granule morphology was observed for treatment with citral; only a few existing wrinkled flattened empty hyphae.

### 2.4. Determination of Cell Membrane Permeability

**Figure 3:**
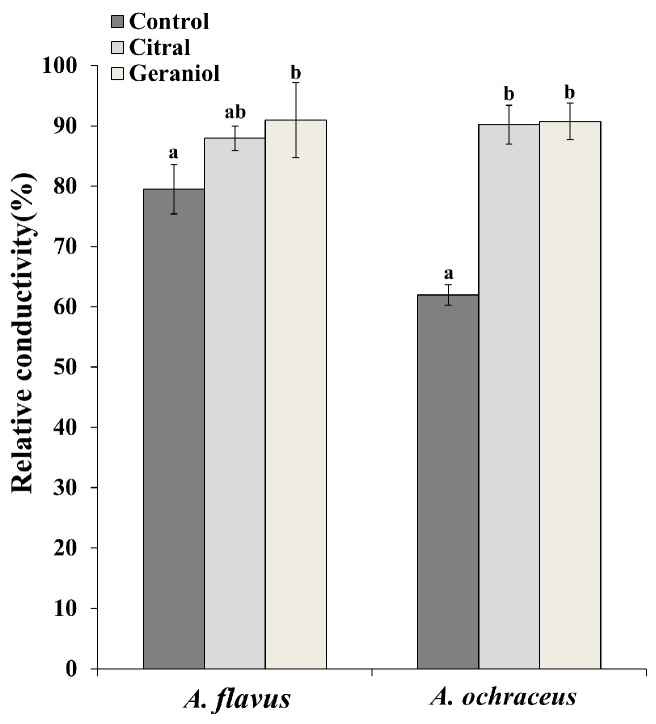
Relative membrane permeability of two toxigenic *Aspergillus* spp. after treatment for 7 days with control, citral, or geraniol. Data are the mean ± SD of three independent experiments. Columns with different letters at the same group indicate significant difference (*p* < 0.05) according to Duncan’s test.

One approach used for determining the mode of action of the antimicrobials against the pathogens was to measure the relative electrical conductivity from the suspensions of *A. flavus* and *A. ochraceus* (Figure 3). The change of electrical conductivity reflected the effect of cell membrane permeability in the pathogens caused by EOC treatment. The treatments with citral and geraniol both resulted in an obvious increase of electrical conductivity in *A. ochraceus*. The conductivity due to electrolyte leakage in *A. flavus* from the control group was 79.4 ± 4.1%, but this value reached 91 ± 6.2% after geraniol treatment (*p* < 0.05). However, there was no apparent change for *A. flavus* when exposed to citral.

### 2.5. Effect of EOCs on Cellular ROS Increase

**Figure 4:**
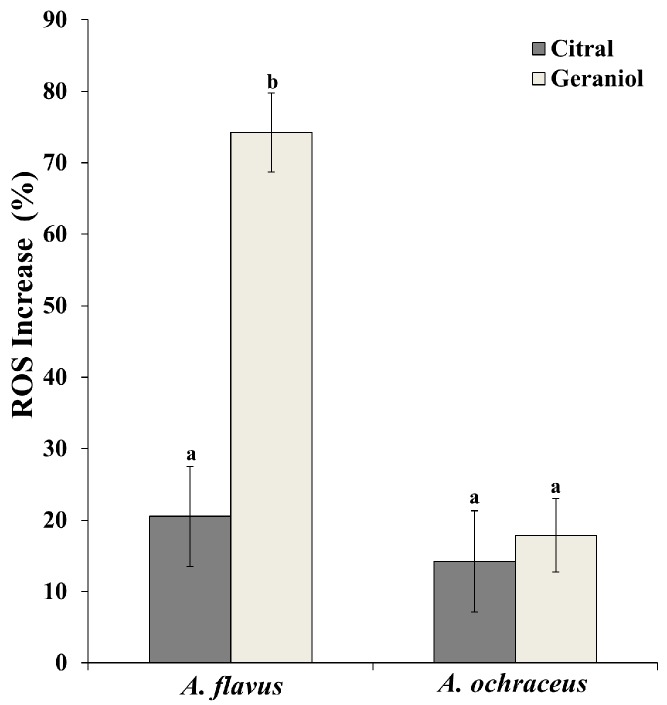
Effects of citral and geraniol on the increase of ROS in two *Aspergillus* spp. Data are the mean ± SD of three independent experiments. Columns with different letters at the same group indicate significant difference (*p* < 0.05) according to unpaired Student’s *t*-test.

ROS production and accumulation in the cell exerts oxidative stress, which may lead to the damage of cellular structures and eventually induce apoptosis [8]. Therefore, we investigated the relationship between the antifungal action of EOCs and oxidative stress in pathogens. The results are demonstrated in Figure 4. The ROS increase was 20.5 ± 7.0% in *A. flavus* and 14.2 ± 7.1% in *A. ochraceus* when treated with citral. Geraniol treatment showed a rapid increase of ROS accumulation in *A. flavus* and reached values of 74.2 ± 5.5%, which indicated that geraniol treatment resulted in an evident stimulation of ROS generation. There was no significant difference in ROS increase in *A. ochraceus* between geraniol and citral.

### 2.6. Gene Expression Analysis

**Figure 5:**
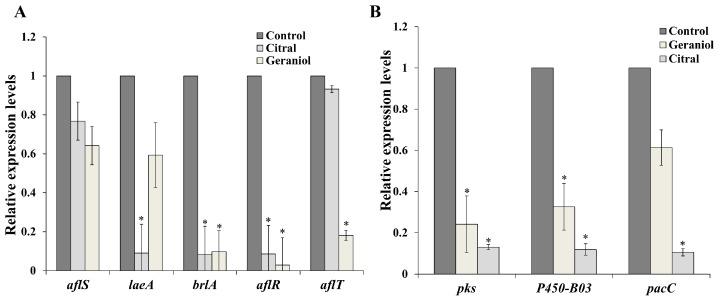
Relative mRNA expression of growth- and secondary metabolism-related genes of *Aspergillus* spp. after treatment with geraniol and citral, by qRT-PCR. (**A**) Expression analysis of growth and aflatoxin biosynthetic genes in *A. flavus*; (**B**) Expression analysis of growth and ochratoxin biosynthetic genes in *A. ochraceus.* Values represent fold changes on the basis of the control (set as 1). Columns with an asterisk (*p* < 0.05) at the same gene were considered differentially expressed if the corresponding fold change was ≥2 or ≤0.5.

For the purpose of revealing the molecular mechanisms behind the inhibitory role of the EOs’ major components in pathogens, some growth-related and secondary metabolite-related genes of *A. flavus* and *A. ochraceus* were monitored by quantitative real-time PCR (qRT-PCR) in mycelia that were treated with EOCs. As indicated in Figure 5A, all growth- and secondary metabolite-related genes in *A. flavus* were downregulated in the geraniol treatment group. Among the five downregulated genes, *aflR*, which is a transcription activator of aflatoxin biosynthesis, was lowly expressed after geraniol treatment. Meanwhile, the key regulatory gene of secondary metabolites, *laeA*, was downregulated with an average 11.1-fold decrease when exposed to citral. In addition, both citral and geraniol resulted in the expression reduction of the sporulation gene *brlA* compared to the control.

### 2.7. Cotreatment with EOCs and MAP for Cereal Preservation

**Figure 6:**
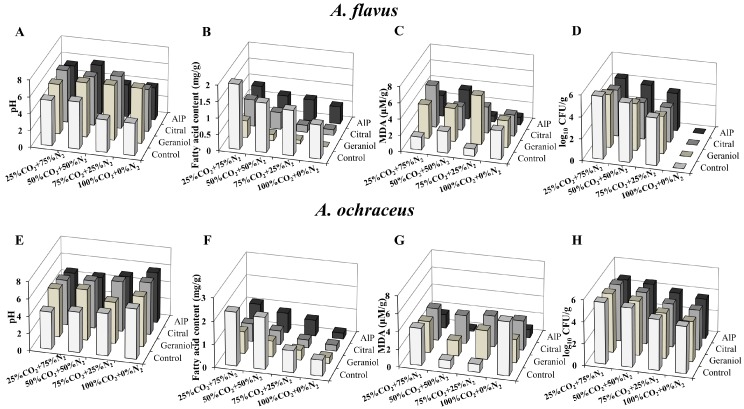
Effects of combined EOCs and MAP on the preservation-related parameters in rice grains. The grains were treated with geraniol (0.5 μL/mL), citral (0.5 μL/mL), AlP (10 mg/L), and sterile water (control) respectively for 8 days under different MAP conditions (25% CO_2_ and 75% N_2_; 50% CO_2_ and 50% N_2_; 75% CO_2_ and 25% N_2_; 100% CO_2_) at 25–30 °C and 80–90% relative humidity (RH). (**A**) Changes in pH values with *A. flavus*; (**B**) Changes in fatty acid content with *A. flavus*; (**C**) Changes in MDA content with *A. flavus*; (**D**) Changes in colony number of *A. flavus*; (**E**) Changes in pH values with *A. ochraceus*; (**F**) Changes in fatty acid content with *A. ochraceus*; (**G**) Changes in MDA content with *A. ochraceus*; and (**H**) Changes in colony number of *A. ochraceus*.

Figure 6 presents the quality characteristics and growth of spoilage organisms on fresh rice with significant synergistic treatments of EOCs and MAP (CO_2_ and N_2_). The pH dropped fast in all MAP conditions without EOCs eight days after inoculation with *A. flavus*. The pH in the control had a fluctuation, but a stable level of pH was observed for *A. ochraceus* with treatment with the combined EOCs and different gas composition. In terms of the content of fatty acids, compared to the untreated control and AlP, the geraniol or citral combined with MAP obviously postponed the increase of fatty acid content whether the grains were challenged with *A. flavus* or *A. ochraceus*. The fatty acid content in geraniol with 100% CO_2_ treatment reached the lowest level of 0.11 NaOH mg/g and was one-tenth of that of the control group. Moreover, fatty acid content had a gradual decline with the increasing proportion of CO_2_ atmosphere composition over eight days when using the same EOCs in each group. With respect to MDA content, no distinct changes for either pathogen were illustrated in the effects of the diversified gas treatments with geraniol or citral as a fumigant. Concurrently, the cotreatment of geraniol or citral with identical MAP manifested higher MDA content in comparison with the sterile water control and AlP positive control. In the light of Figure 6D–H, the combined EOCs and MAP effectively delayed the growth of *Aspergillus* spp. on grains after eight days’ exposure when compared to the single MAP treatment. In particular, the EOC treatments together with 100% CO_2_ gas packaging nearly inhibited *A. flavus* growth in grains. In addition, packaging with gas components of 50% CO_2_:50% N_2_ and 75% CO_2_:25% N_2_ associated with citral denoted a better antifungal effect against *A. ochraceus* than AlP, and their reduction levels were 4.78 log_10_ colony forming unit (CFU)/g and 4.17 log_10_ CFU/g, respectively. However, there were no obvious differences in the effect of the other gas compositions.

## 3. Discussion

Cereals represent the most important source of food samples in many countries. They have been highly susceptible to pathogens and their secretion of mycotoxins due to the disturbance or aging of tissue physiology and mechanical damage preharvest and postharvest, which not only causes large pecuniary losses, but also a serious threat to human health. Recently, the use of a number of natural-product-derived volatile chemicals in crops and food for the treatment of diverse agricultural diseases has drawn widespread attention from researchers. Polyphenols, vitamins, alkaloids, saponins, polysaccharides, and bioactive peptides derived from many different plants have displayed antioxidant capabilities and antimicrobial activities. Plant EOs and related EOCs, odorous and volatile products with diversified bioactive functions formed by secondary metabolism, have been applied in medicine and as food additives, such as flavor enhancers, aromatizers, and preservatives.

In this study, we tested about 30 plant EOs (Supplementary Table S2) and their major compounds to evaluate their antifungal activity against two typical pathogens of grains (*A. flavus* and *A. ochraceus*) by fumigation. In solid media, only seven vaporous EOCs exhibited an inhibitory effect against the above molds (Supplementary Table S1), and two EOCs (citral and geraniol) with better properties were appropriate for subsequent studies. The impact of these two EOCs on fungal growth was increased progressively with concentration. Interestingly, *A. ochraceus* with treatment of geraniol at the concentration of 0.3 to 0.6 μL/mL was not completely inhibited, owing to the saturation of geraniol in the vapor phase.

Based on the previous results about the inhibitory effect of EOCs in vitro, we decided to choose the best antifungal EOCs for in situ experiments. The pH value, fatty acid content, MDA content, and colony number parameters of the grain were tested to make a comprehensive and systematic evaluation of grain quality after EOC treatment. The pH declined as storage time increased, which was accounted for by the reason that the two EOCs were slightly acidic. A recent study declared that the decreasing pH was mainly caused by the degradation of proteins and metabolism of pathogens in rice, and the pH balance in cells would improve the nitrogen use efficiency of rice to extend storage life [9]. EOCs with low pH values in foodstuffs were more hydrophobic and had more affinity to the target hydrophobic areas of proteins in the bacterial cell membrane [10,11]. Therefore, the lower pH of grain may help the EOCs play a better inhibitory effect against pathogens. With respect to fatty acid content, the increase of lipid acidity level being an early indicator of mold spoilage, some toxigenic fungus produced lipase in suitable conditions, which accelerated the hydrolysis of rice fats and caused an increase the fatty acid content of grain [12]. Our results demonstrated that citral significantly postponed the ascent of fatty acid content. MDA is the ultimate product of membrane lipid peroxidation in rice plants, and the cell undergoes oxidative stress with the increase of lipid peroxidation level and the accumulation of MDA [13]. In this study, the EOCs could maintain the lower level of MDA to protect the rice from oxidative damage. Concerning the microbial numbers, these parasitic microbes competed with the grains for oxygen and derive nutrients from grain tissues for their growth to deter grain respiration [14,15]. Besides, there are many factors, such as temperature, humidity, pH, and microorganisms, which consistently impact the efficacy of the combined biofungicides in the field experiments. Thus, the major compounds of EOs at the MIC in vitro used in grain could not realize the complete inactivation of fungi in situ. SEM analysis signified that both geraniol and citral effectively protected starch granules from the challenge of *A. flavus* and *A. ochraceus*. It was previously found that carvacrol was very effective when used against the foodborne pathogen *Bacillus cereus* at 0.15–0.75 μL/g on boiled rice [16], which indicated that EOCs could be used in food or agricultural products to achieve potential antibacterial activity.

The mode of action of EOCs for microbes could be attributed to the changing permeability of cell membranes and walls, as well as to the DNA, to alter gene expression, cellular respiration, and energy metabolism and eventually kill the cells [3]. In our current study, we explored citral and geraniol as bioactive agents against *A. flavus* and *A. ochraceus* for grains and the underlying antifungal mechanisms. *A. flavus* and *A. ochraceus* were completely inhibited at the MIC of citral and geraniol in vitro, at which growth and metabolism of *Aspergillus* spp. could not be detected; therefore, we adopted lower concentrations of citral and geraniol for use with the two pathogens to better expound the antifungal action of the EOs’ major components. The modes of antifungal action of citral and geraniol on the common *Aspergillus* spp. of grain were investigated by evaluating cell membrane permeability, cellular ROS change, and growth and secondary metabolite gene expression. According to the results for relative conductivity, both EOCs showed a better capability of increasing cell membrane permeability in *A. ochraceus* compared to *A. flavus*, which led to the leakage of intracellular constituents. It was likely that the leakage was caused by the interaction between antimicrobial ingredients and the cytoplasmic membrane [17]. The different relative conductivity changes of *A. ochraceus* and *A. flavus* might be due to the involvement of different components and binding sites of the cell membrane when treated with the same EOC. Meanwhile, the electrical conductivity of *A. ochraceus* increased by 28.3% and 28.8% after exposure to citral and geraniol, respectively, indicating that the integrity of the cell membrane was partially disrupted. When damage to the cell membrane occurred, more small molecules such as electrolytes were released, causing the higher electrical conductivity to accelerate cell death [18].

Oxidative stress is considered as a signal for inducing cell death in response to various external environmental stresses and internal psychophysiological stimulation. ROS, mainly generated in the mitochondria through the oxidative process, are a series of intermediate products formed during the process of oxygen metabolism, and excessive ROS attack cellular biomolecules such as lipids, proteins, and DNA to cause irreversible oxidative damage [19]. This study indicated that citral and geraniol induced rapid and significant ROS production in *Aspergillus* spp. (Figure 4). This result was in agreement with a previous report which showed that citral and carvacrol caused oxidative damage in *Escherichia coli* [20]. Besides, it was inferred that ROS formation was the main factor for the inhibition of *A. flavus* by geraniol, according to a 74.2% ROS increase. Previous investigations suggested that high ROS levels reduced intracellular antioxidants, impaired DNA and proteins, and thus triggered cell damage or apoptosis [21]. The mechanism of microbial inactivation by ROS assumed that ROS involved the formation of Fenton-mediated hydroxyl radicals through the tricarboxylic acid (TCA) cycle and ultimately initiated cell death [22]. The accumulating evidence implied that the geraniol swiftly facilitated the induction of more ROS in *A. flavus*, which was similar to a prior study which described that cinnamaldehyde inhibited *A. flavus* by interfering with the intracellular redox system to affect metabolic regulation [23].

To reveal the inhibitory effects of citral and geraniol on *A. flavus* and *A. ochraceus* from a molecular perspective, some key genes which were closely associated with growth and toxin metabolism were detected. The expression profile of key regulatory genes of aflatoxin biosynthesis and growth in *A. flavus* were analyzed (Figure 5A). Among these genes, the transcriptional activator gene of asexual sporulation *brlA* and the global regulatory gene of secondary metabolite *laeA* were both highly downregulated by citral. Previous research demonstrated that disruption or deletion of the *brlA* gene resulted in loss of the ability for premature development in *Aspergillus* spp. [24]. It was elucidated that citral modulated growth-related genes of *A. flavus* to achieve its antifungal property. Interestingly, we observed that the citral and geraniol not only influenced the growth regulatory genes, but also had inhibitory capacity for the expression of secondary metabolism-related genes, especially for aflatoxin biosynthetic genes, which signified that citral and geraniol had sufficiently inhibited them to prevent aflatoxin from being synthesized in addition to retarding *A. flavus* growth at low concentrations. The *aflS* gene is a pathway regulator, and *AflR* is a transcriptional activator of aflatoxin pathway cluster structural genes [25]. When *A. flavus* was in an appropriate condition, *AflS* bound to *AflR* to form a complex and seek a promoter region for aflatoxin biosynthesis [26]. Thus, in our study, it was found that citral and geraniol could inhibit major biosynthetic genes. This is consistent with previous reports, which manifested that cinnamaldehyde, citral, and eugenol inhibited aflatoxin production in *A. parasiticus* by downregulating the aflatoxin pathway genes (*aflR*, *aflT*, *aflD*, *aflM*, and *aflP*) [27]. Meanwhile, aflatoxin accumulation was achieved in the context of sporulation. It was suggested that citral and geraniol resulted in mycotoxin prevention by inhibiting the sporulation gene *brlA*.

Concerning the transcriptional level in *A. ochraceus*, although the growth and metabolite biosynthesis of *A. ochraceus* at the molecular level have not been completely elucidated, some of critical genes have been strongly correlated with metabolite pathways. *PacC* is a pH-dependent, alkaline-induced regulator responsible for growth in *A. ochraceus* [28]. In this study, the *pacC* gene was effectively induced at a transcriptional level in *A. ochraceus* treated by citral, which explicated the mode of action of citral against *A. ochraceus*. Nevertheless, geraniol treatment appeared to have no apparent inhibitory effect on *pacC*. A different mode of action for geraniol might be illustrated. Additionally, ochratoxin, as a secondary metabolite, was a polyketide mycotoxin mainly produced by *Aspergillus* spp. and *Penicillium* spp., and was classified as a group 2B potential carcinogen by the International Agency for Research on Cancer (IARC) [29]. Ochratoxin biosynthesis is dependent on enzymatic reactions because of its special molecular structure. Thence, *pks* and *p450-B03*, as two key genes which encode a polyketide synthase and a cytochrome p450 monooxygenase, respectively, have played a vital role in the ochratoxin biosynthesis of *A. ochraceus* [30,31]. In line with these findings, qRT-PCR analyses denoted that both citral and geraniol had negative regulation effects on *pks* and *p450-B03* to inhibit the ochratoxin production. These results were supported by the evidence which revealed that clove, cinnamon, lemongrass, and mandarin EOs suppressed the relative expression of the selected ochratoxin-related genes [32]. Nevertheless, some earlier works showed that citrus EOs and their major components exerted inhibitory effects on fungal growth, but not on toxin accumulation, and even induced the higher production of ochratoxin in *A. westerdijkiae* [33,34]. This is not inexplicable, given that EOCs with low doses might create stress conditions and stimulate the enhancement of secondary metabolism in fungi for resisting external pressures. Previous research also demonstrated that geraniol interfered with bilayer fluidity and caused genotoxicity in some fungi [35,36], which was further confirmed to be a consistent explanation of the antifungal mode of specific EOCs.

There is no doubt that the antifungal activity of EOCs can be attributable to multiple mechanisms because of the complexity of fungal cell structure and composition. However, not all of these mechanisms are involved in fungal inactivation and they might have many intricate causal or feedback relationships. In accordance with our study, citral exhibited antifungal activity mainly by diminishing the sporulation- and growth-related genes for *A. flavus* and *A. ochraceus*. The geraniol displayed inhibitory effectiveness against *A. flavus* predominantly by increasing the intracellular ROS accumulation and showed toxicity against *A. ochraceus* principally by impairing the cell membrane.

EOC volatiles appear to be promising control methods against microorganisms in packaging. The majority of the work initiated so far has concentrated on the control effects of EOCs or MAP single treatments against pathogen contamination in grain. In our study, EOCs in combination with MAP administered to rice displayed weakened grain respiration with less loss of EOCs in the vapor phase, achieving the better preservation effect. MAP (100% CO_2_) showed complete inactivation for *A. flavus*, with <1.0 log_10_ CFU/g after eight days of storage. Owing to *A. flavus* being an aerobic fungus, high-purity CO_2_ could exert an antifungal effect by affecting the enzymatic decarboxylation of *A. flavus* [37]. However, the 100% CO_2_ treatment of grain is impractical during industrial grain storage because of high cost and high energy consumption. The lower level of CO_2_ combined with EOCs should be taken into consideration.

AlP is the chemical fumigant commonly used to control the corruption of grain; however, PH_3_ released by AlP might be harmful for human health. Based on our results, citral or geraniol cotreatment with MAP significantly alleviated spoilage in grain and preserved grain quality to a maximum without any chemical residue, as opposed to AlP treatment, which indicated that the EOs’ major components as a novel potential approach could displace the conventional chemical fumigant for future use in grain preservation. Additionally, EOCs combined with MAP had better effects on reducing colony number and improving grain quality than EOC or MAP treatments alone. Therefore, a low dose of EOCs combined with modern preservation technology such as low temperature and MAP could not only reduce the growth of pathogens for extending the shelf life of agricultural products, but also maintain sensory quality.

## 4. Materials and Methods

### 4.1. Chemical and Reagents

Materials: The fresh rice grains were purchased from a local market in the Xihu district of Hangzhou, China. PDA medium (potato, 200 g; dextrose, 20 g; agar, 15 g; and distilled water, 1000 mL; pH 5.6 ± 0.2) and plate count agar (PCA) medium (pancreatic digest of casein, 5.0 g; yeast extract, 2.5 g; dextrose, 1.0 g; agar, 15 g; and distilled water, 1000 mL; pH 7.0 ± 0.2) medium were autoclaved at 121 °C for 20 min. Tablets of aluminium phosphide (AlP) was obtained from Shanghai Skyblue Chemical Co., Ltd. and stored in a dry safety cabinet. EOs’ main bioactive components such as geraniol and carvone which were extracted and separated from natural *Lippia rugosa* EO and caraway EO, respectively, were obtained from Aladdin Reagent Co., Ltd. (Shanghai, China). Cinnamaldehyde of *Cinnamomum zeylanicum* Blume EO’s component, eugenol of clove EO’s component, and 2′,7′-dichlorofluorescin diacetate (DCFH-DA) were purchased from Sigma-Aldrich (St. Louis, MO, USA). Citral and anethole were extracted from lemongrass EO and anise EO with hydrodistillation to attain a high-purity grade (95–100%) by anhydrous sodium sulphate drying in accordance with a previously described method [38]. The active compounds from other EOs were available from the respective manufacturers. All plant EOCs were stored at 4 °C for further testing and their corresponding MICs against target microbes are presented in Supplementary Table S2. All other chemical reagents, including trichloroacetic acid (TCA), thiobarbituric acid (TBA) glutaraldehyde, osmium (VIII) oxide (OsO_4_), and Tween 20 were of analytical grade.

### 4.2. Strains and Culture Conditions

Two toxigenic fungi were used: *Aspergillus ochraceus* CICC 2471 was purchased from China Center of Industrial Culture Collection (CICC), and *Aspergillus flavus* NRRL3357 was supplied by Institute of Biotechnology in the College of Agriculture and Biotechnology of Zhejiang University (Hangzhou, China). All strains were preserved with 25% (*v*/*v*) glycerol solution at −80 °C, were periodically subcultured in PDA slants at 4 °C, and were cultivated on PDA medium at 25 °C for 5 days before use in this study.

### 4.3. In Vitro Antifungal Activity Assay

The divided-plate method to determine antifungal activity was used as described by Chaves et al. [39] with some modifications. Briefly, a fresh 5 mm plug of pathogens was transferred to the center of one half of a divided plate containing PDA medium, and each essential oil with a different volume was added on another compartment of the plates (50 mL) to attain 0 (control), 0.1, 0.2, 0.3, 0.4, 0.5, and 0.6 μL/mL concentrations. Then, the plates were hermetically sealed with parafilm to prevent the escape of oil volatiles. The plates were incubated at 25 °C for 7 days, and the radial growth diameters of colonies were measured in two mutually perpendicular directions to obtain the mean diameter each day. These measurements were carried out three times. The lowest concentration of essential oil which completely inhibited fungal growth was designated as the minimum inhibitory concentration (MIC), and the percent of mycelial inhibition was calculated using the following equation: (colony diameter in control − colony diameter in treatment)/(colony diameter in control − 5) × 100%, where 5 is the initial diameter of the plaque (mm).

### 4.4. In Situ Investigation with EOC Fumigation against Pathogens of Grains

Based on results of the in vitro antifungal test, geraniol and citral were selected for further study. The fresh rice grains from a local supermarket (Hangzhou, China) were stored at 4 °C before use. The grains were sterilized with the 0.1% (*v*/*v*) sodium hypochlorite solution for 1 min, washed with sterile water, and dried for 30 min in vertical clean benches at room temperature. Aliquot samples of 60 g were placed in sterilized Petri dishes (9 cm) and transferred to the porcelain plate of a disinfected desiccator (inner diameter: 300 mm, height: 370 mm, volume: 1.9 L). Spores of the pathogen were harvested from the PDA medium and suspended in sterilized phosphate buffer (pH 7.2) to 1.0 × 10^4^ conidia/mL concentration with serial dilution by haemocytometer count. Then, 200 μL of conidial suspension was evenly inoculated on the grains. An autoclaved filter paper disc (6 mm) was laid on the bottom of the desiccator with the addition of 950 μL of geraniol and citral solution, respectively. Control was performed with the same volume of sterile distilled water on the disc. AlP tablets (19 mg) were used as the positive control. Subsequently, the lid was smeared with Vaseline and covered on the desiccator to avoid the leakage of EOCs. The desiccators were stored on an artificial climate chest at 25–30 °C and 80–90% relative humidity (RH) for 0, 2, 4, 6, and 8 days. Each experiment was done with three repetitions.

### 4.5. Determination of pH, Fatty Acid Content, MDA Content, and Colony Number

Grains of rice treated with the geraniol and citral were respectively collected 0, 2, 4, 6, and 8 days after inoculation. Then, these grains were ground into powder and passed through 80 mesh sieves for the following measurements. Five grams of rice powder was dissolved at the rate 1:10 (*w*/*v*) in distilled water to record pH by precision digital pH meter (PB-10, Sartorius Stedim Biotech, Goettingen, Germany). Fatty acid contents of the grains were assessed by referring to standard methods [40], and test results were expressed as sodium hydroxide equivalents. The MDA was measured using a published method [41] with some slight modifications. Briefly, 1 g of ground sample was extracted with 4 mL 10% (*w*/*v*) trichloroacetic acid (TCA), and then the sample extract was centrifuged at 6000 g at 4 °C for 10 min. The supernatant was added to 4 mL thiobarbituric acid (TBA). The mixture was heated in a boiled water bath for 15 min and was cooled with running water. The supernatant was collected by centrifugation at 1000 g for 10 min and was measured at 532 nm of absorbance by using a microplate multimode spectrophotometer (SpectraMax M5, Molecular Devices, San Francisco, CA, USA). The MDA content was calculated and normalized to micromole per gram (μmol/g). For the detection of colony number, 5 g of sample was aseptically transferred to an autoclaved Erlenmeyer flask containing 45 mL of sterile distilled water and homogenized in a Stomacher blender (Ultra-Turrax T25, IKA Laboratory Technology, Staufen, Germany) for 5 min. Afterwards, the homogenate was diluted serially 10-fold in sterile distilled water and a 200 μL aliquot of diluent was mixed with 15 mL of 40 °C PCA medium on a sterilized Petri dish. After the liquid medium of the dish had solidified, the plates were incubated at 28 ± 2 °C for 36–48 h to observe and count visible microbial colonies.

### 4.6. SEM Analysis

The surface morphology and microstructures of grains were characterized by a field emission SEM (TM-1000, Hitachi, Japan) at an acceleration voltage of 15 kV. The following procedures were adopted for the samples before microscopic observation. The control and treated grains were first soaked in phosphate buffer (0.1 M; pH 7.0) containing 2.5% (*v*/*v*) glutaraldehyde for 4 h, washed with phosphate buffer three times, and then postfixed with 1% (*w*/*v*) OsO_4_ in phosphate buffer for 1 h. After that, the samples were dewatered by a graded series of ethanol (50–100%, 10% intervals) for 20 min and dehydrated in a critical point dryer (HCP-2, Hitachi, Japan) with liquid CO_2_. Finally, the dried specimens were coated with Pt–palladium for SEM examination.

### 4.7. Permeability of the Toxigenic Cell Membrane Assay

Geraniol and citral were separately mixed with a small amount of sterile 0.01% (*v*/*v*) Tween 20 to prepare the EOC emulsions. Subsequently, the EOC emulsions and the control, respectively, were added into the 50 mL of PDB broth to a final concentration of 0.02% (*v*/*v*). Simultaneously, 200 μL of 10^6^ conidia/mL suspension of the two pathogens, *A. flavus* and *A. ochraceus*, were inoculated into each broth, respectively. The same amount of pathogen inoculum was cultured in the media without adding EOC emulsions to serve as the control. They were incubated in the dark at 28 °C on a rotary shaker (QYC 2102, FUMA, Shanghai, China) at 200 rpm. On day 7, the supernatant was examined for cell permeability after centrifugation at 4000 g for 15 min. The fungal mycelia were harvested by filtering and washed with sterilized water for subsequent ROS assay and RNA extraction. The cell permeability of pathogens was measured by the method described previously [42] with some modifications. The supernatant was detected by conductivity meters (DDS-307, INESA Instrument, Shanghai, China). The corresponding mycelia were heated for 20 min in a boiling water bath to determine the electric conductivity. The PDB without EOCs and pathogens was used for the initial electric conductivity.

The relative conductivity was calculated as

(1) $$\mathrm{Relative}\;\mathrm{conductivity}\;\left(\%\right)=\frac{C1-C0}{C2-C1}\times 100$$

where *C*0 is the initial electrical conductivity (μs/cm), *C*1 is the electrical conductivity of the supernatant (μs/cm), and *C*2 is the electrical conductivity of the supernatant after 20 min in boiling water bath (μs/cm).

### 4.8. Determination of ROS in Pathogens

Intracellular production of ROS in pathogens was measured using DCFH-DA (2′,7′-dichlorodihydrofluorescein diacetate) probe. The fungal mycelia were obtained as described in Section 4.7 and were incubated with 20 µM DCFH-DA at 30 °C for 30 min in a dark environment. The hyphae were washed with phosphate buffer (0.1 M; pH 7.0) twice and were disrupted in the same buffer with acid-washed glass beads (425–600 μm diameter) by tissue homogenizer (Tissuelyer-48, Jingxin Technologies, Shanghai, China) for 3 min. The mixture was centrifuged at 10,000 g, 4 °C for 5 min. The supernatant was analyzed at λ_excitation_ = 485 nm and λ_emission_ = 535 nm by a fluorescence microplate reader (Spark 10M, Tecan, Mannedorf, Switzerland).

### 4.9. Isolation of Total RNA and cDNA Synthesis

Fungal mycelia were collected by filtering and ground under liquid nitrogen conditions to extract the total RNA by using the MiniBEST Plant RNA Extraction Kit (Takara, Otsu, Japan) according to the manufacturer’s instructions. Then, the obtained RNA was evaluated by a nucleic acid spectrophotometer (Nanodrop ND-1000, Thermo Scientific, Waltham, MA, USA) at 260 nm and 280 nm to check the purity and integrity. The RNA samples were treated with gDNA Eraser (Vazyme, Nanjing, Jiangsu, China) to avoid interference from the gDNA and the synthesis of complimentary DNA (cDNA) was carried out using HiScript^®^ II QRT SuperMix Kit (Vazyme, Nanjing, Jiangsu, China).

### 4.10. Quantification of Gene Expression by qRT-PCR

The qRT-PCR experiment was conducted with a 20 μL reaction volume from 2 μL of cDNA (corresponding to 1 μg RNA) in triplicate using ChamQ™ SYBR^®^ qPCR Master Mix (Vazyme, Nanjing, Jiangsu, China), and the fluorescent signal was captured by the QuantStudio3 Real-Time PCR System (Applied Biosystems, Thermo Scientific, Waltham, MA, USA). Primers used in this study are listed in Supplementary Table S3. The qRT-PCR procedure included initial denaturation at 95 °C for 30 s, followed by 40 cycles of 10 s denaturation at 95 °C, 30 s annealing at 60 °C, and 15 s extension at 72 °C. β-tubulin and GADPH were used as the endogenous controls for *A. flavus* and *A. ochraceus*, respectively. The results were expressed as the fold change of relative gene expression on the basis of the method of 2^−ΔΔCT^ [43] compared to the control samples.

### 4.11. Synergistic Effect of EOCs Combined with MAP against Pathogens on Grains

The fresh rice grains were pretreated and toxigenic spore suspensions were prepared as aforementioned in Section 4.4*.* Twenty-five-gram grain samples with 200 μL of conidial suspension inoculation were placed in sterilized Petri dishes (6 cm) and transferred into UV-sterilized polyethylene terephthalate (PET) containers (20 cm × 12 cm black boxes). An autoclaved piece of disc pipetted with essential oil separately was added into each container. The treatments were designated as follows: (A) geraniol; (B) citral; (C) AlP (positive control); and (D) sterile distilled water (negative control). Thereafter, these containers were packaged under various modified atmospheres with a MAP machine (DT-6D, Dajiang Intelligent Equipment, China) and were instantly heat-sealed by a vacuum sealer. The different modified atmospheres were appointed as follows: (I) 25% CO_2_ and 75% N_2_; (II) 50% CO_2_ and 50% N_2_; (III) 75% CO_2_ and 25% N_2_; and (IV) 100% CO_2_. Thereafter, these packing boxes were stored on an artificial climate chest at 25–30 °C and 80–90% relative humidity (RH) for 8 days. Three replicate treatments were considered for each experiment.

### 4.12. Statistical Analysis

All experiments were repeated three times and all quantitative results were analyzed to express as the mean ± standard deviation (SD). Data were subjected to one-way analysis of variance (ANOVA) and the means were evaluated by Duncan’s multiple range tests or unpaired Student’s *t*-test to determine significant differences (*p* < 0.05) using SPSS statistics 23 software (IBM Co., Chicago, IL, USA). All figures were plotted with the GraphPad Prism 7 (GraphPad Software Co., La Jolla, CA, USA).

## 5. Conclusions

The present study demonstrated that two EOCs (citral and geraniol) have antimicrobial effects against two pathogens (*A. flavus* and *A. ochraceus*) in vitro and in grain, and explored their mode of action. The results indicated that citral and geraniol could apparently inhibit fungal growth at certain concentrations by changing cell membrane permeability, inducing intracellular accumulation of ROS, and interfering with key related genes. Furthermore, the citral and geraniol in combination with MAP could have synergistic effects on controlling *Aspergillus* spp. infection of grain, which presents an alternative preservation method to improve the shelf life of grain by using natural bioactive substances. Collectively, these EOCs could provide a basis for the development of novel grain preservatives for practical applications.

## Figures and Tables

**Table 1:** Antifungal effects of citral and geraniol in different concentrations in the vapor phase against toxigenic fungi on potato dextrose agar (PDA) medium.

EOC Concentration		*A. flavus*		*A. ochraceus*	
(μL/mL)		Growth (mm) ^1^	Inhibition Rate (%) ^1^	Growth (mm) ^1^	Inhibition Rate (%) ^1^
Citral	0	65.67 ± 3.01a	0.00 ± 0.00a	66.83 ± 1.26a	0.00 ± 0.00a
	0.1	7.03 ± 0.58b	96.64 ± 0.26b	6.68 ± 0.29b	97.28 ± 0.96b
	0.2	6.33 ± 0.22b	97.80 ± 0.37b	6.00 ± 0.09bc	98.38 ± 0.17c
	0.3	6.00 ± 0.16b	98.35 ± 0.18c	5.28 ± 0.95cd	99.54 ± 0.35c
	0.4	5.52 ± 0.10b	99.14 ± 0.48d	5.00 ± 0.00d	100 ± 0.00d
	0.5	5.00 ± 0.00b	100 ± 0.00b	5.00 ± 0.00d	100 ± 0.00e
	0.6	5.00 ± 0.00b	100 ± 0.00b	5.00 ± 0.00d	100 ± 0.00e
Geraniol	0	65.67 ± 3.01a	0.00 ± 0.00a	66.83 ± 1.26a	0.00 ± 0.00a
	0.1	8.87 ± 0.12b	93.62 ± 0.35b	8.47 ± 0.58b	94.39 ± 0.16b
	0.2	7.03 ± 0.59bc	96.64 ± 0.26c	6.37 ± 0.12c	97.79 ± 0.23c
	0.3	6.00 ± 0.13c	98.35 ± 0.38d	6.00 ± 0.16c	98.38 ± 0.13d
	0.4	6.00 ± 0.03c	98.35 ± 0.08d	6.00 ± 0.32c	98.38 ± 0.25d
	0.5	5.95 ± 0.70c	98.44 ± 0.52d	6.00 ± 0.24c	98.38 ± 0.06d
	0.6	5.95 ± 0.35c	98.44 ± 0.05d	6.00 ± 0.15c	98.38 ± 0.11d

^1^ Plaque diameters were measured at the 7th day after inoculation. In the table, the values are expressed as mean ± SD (n = 3). Data in the same column with different lower-case letters indicate a significant difference at *p* < 0.05 by Duncan’s test.

## Supplementary Materials

The tables including the inhibitory activity of EOCs and plant EOs against *Aspergillus* spp. and primers for this study are available in the supplementary files online.
