# Supplementary material for: Antifungal Activity of Essential Oil Compounds (Geraniol and Citral) and Inhibitory Mechanisms on Grain Pathogens (Aspergillus flavus and Aspergillus ochraceus)
Source: Molecules. 2018 Aug 22;23(9):2108. doi: 10.3390/molecules23092108 (PMC6225121; doi:10.3390/molecules23092108)
Supplement: Supplementary file 1 [file molecules-23-02108-s001.pdf]

Table S1. Mycelial growth (mm) of pathogens on PDA medium with various vaporized EOCs

Pathogens	EOCs	Growth with different times (day)[1]							Inhibition rate (%)[1]
		1	2	3	4	5	6	7	
A. flavus	Control	7.17 ± 0.14a	15.17 ± 0.2a	26.42 ± 3.13a	42.67 ± 1.38a	51.58 ± 2.40a	57.58 ± 2.40a	65.67 ± 3.01a	0.00 ± 0.00a
	Geraniol	5.58 ± 0.14c	5.58 ± 0.14e	5.67 ± 0.14e	5.75 ± 0.25e	5.75 ± 0.25f	5.83 ± 0.29f	5.83 ± 0.29e	98.61 ± 0.52e
	Eugenol	6.92 ± 0.14a	14.67 ± 0.29b	23.08 ± 0.76b	28.75 ± 2.38c	33.00 ± 2.61d	35.42 ± 2.70d	38.50 ± 4.77d	44.45±10.56d
	Citral	5.67 ± 0.14c	5.67 ± 0.14e	5.92 ± 0.29e	6.08 ± 0.14e	6.42 ± 0.29ef	6.50 ± 0.10f	7.33 ± 0.80e	96.18 ± 1.16e
	Cinnamaldehyde	7.08 ± 0.14a	14.75 ± 0.25b	23.58 ± 0.76b	39.50 ± 0.25b	47.42 ± 3.33b	53.00 ± 3.68b	58.50 ± 6.14b	11.65 ±11.01b
	Carvone	6.33 ± 0.14b	6.92 ± 0.14d	8.25 ± 0.75d	9.17 ± 0.88d	9.58 ± 0.95e	11.08 ± 0.95e	11.33 ± 1.13e	89.48 ± 2.40e
	Anethole	6.25 ± 0.43b	12.25 ± 0.25c	19.00 ± 0.43c	28.92 ± 1.13c	37.75 ± 1.52c	44.00 ± 1.73c	51.58 ± 0.14c	23.09 ± 3.67c
A. ochraceus	Control	7.93 ± 0.12a	19.77 ± 0.25a	31.00 ± 1.00a	41.67 ± 1.53a	53.17 ± 2.75a	57.67 ± 2.52a	66.83 ±1.26a	0.00 ± 0.00a
	Geraniol	6.00 ± 0.12c	6.00 ± 0.18e	6.00 ± 0.16f	6.00 ± 0.29f	6.00 ± 0.27e	6.00 ± 0.19e	6.00 ± 0.22e	98.38 ± 0.03e
	Eugenol	6.23 ± 0.25bc	6.77 ± 0.25d	9.00 ± 1.32d	9.17 ± 0.76d	10.17 ± 2.47d	11.50 ±2.18de	10.6 ± 2.47de	90.83 ±3.97de
	Citral	6.00 ± 0.32c	6.00 ± 0.24e	6.00 ± 0.22f	6.00 ± 0.05f	6.00 ± 0.14e	6.00 ± 0.16e	6.00 ± 0.21e	98.38 ± 0.03e
	Cinnamaldehyde	6.07 ± 0.12c	6.93 ± 0.12d	7.67 ± 0.58e	8.67 ± 1.04de	10.00 ± 2.29d	13.67 ± 5.13d	15.00 ± 5.57d	83.95 ± 8.72d
	Carvone	6.17 ± 0.29bc	6.17 ± 0.29e	6.33 ± 0.29f	6.83 ± 0.29ef	7.33 ± 0.76de	10.17 ±3.01de	15.17 ± 3.75d	83.60 ± 5.94d
	Anethole	6.07 ± 0.12c	8.33 ± 0.29c	15.00 ± 1.00c	20.17 ± 1.04c	29.17 ± 1.26c	40.83 ± 1.04c	46.17 ± 1.26c	33.42 ± 1.78c
	Cineole	6.47 ± 0.31b	10.67 ± 0.58b	20.00 ± 0.50b	30.5 ± 2.18b	40.33 ± 1.53b	50.33 ± 1.53b	59.17 ± 1.04b	12.35 ± 3.40b

[1] The values are expressed as mean ± SD (n = 3). Data in the same column with different lowercase letters at each time point indicate significantly different at $p < 0.05$ by Duncan's test.

Table S2. Major bioactive components of plant EOs and their relevant antimicrobial effects

Plant EOs	Source and Distrution	Major Bioactive Components	Target Microbes	Concentration	References
Amomum tsao-ko EO	*Amomum tsao-ko* flower heads	1, 8-cineole (45.24%)<br>rho-propylbenzaldehyde (6.04%)	*Trycophyton mentagrophytes*, *Bacillus subtilis*, *Staphylococcus aureus*, *Escherichia coli*, and *Proteus vulgaris*	0.075%	[1]
Amomum kravanh EO	*Amomum kravanh*	1, 8-cineole (68.42%)<br>α-pinene (5.71%)	*Bacillus subtilis* and *E. coli*	0.05%	[2]
Turmeric leaves EO	*Turmeric* leaves	α-tumerone (35.17%)<br>tumerone (11.93%)<br>β-sesquiphellandrene (11.5%)	*A. flavus*	1.0%-1.5%	[3]
Zingiber officinale EO	*Zingiber officinale* roots	α-zingiberene (36.9%)<br>β-sesquiphellandrene (15.3%)<br>β-bisabolene (8.8%)	*Fusarium moniliforme*, *Penicillium* spp. and *A. niger*	0.064%	[4]
Curcuma longa L. and *Zingiber officinale Rosc* EO	*Curcuma longa L. Zingiber officinal Rosc* fruits	γ-terpinene (40.92%)<br>p-cymene (27.93%)<br>cumin aldehyde (21.20%)	*A. flavus, A. oryzae, A. niger* and *Alternaria alternata*	0.2%	[5]
Foeniculum vulgare L. EO	Fennel leaf	trans-anethole (31-36%)<br>α-pinene (14-20%)<br>limonene (11-13%)	*A. flavus* and *A. paraiticus*	0.001%-0.00125%	[6]
Mint EO	Mint aerial parts	isomenthone (0.2-77.5%)<br>pulegone (1.0-50.6%)<br>piperitone (0.3-13.4%)	*Staphylococcus aureus*, *Pseudomonas aeruginosa*, *E. coli*, *Saccharomyces cerevisiae*, *Monascus*, *Rhizopus*, and *A. niger*	1.49%	[7, 8]
Pepper EO	Pepper fruits	limonene (40.34%)<br>α-phellandrene (24.47%)	Some typical gram-positive bacteria (*Staphylococcus aureus*, *Gambogic section coli* and *E. coli*)	0.75%	[9]

Clove EO	Clove bud	eugenol (71.80%)	*Staphylococcus aureus*, *Bacillus subtilis* and *A. niger*	0.002%-0.013%	[10]
Anise EO	Anise	trans-anethole (88.49%)<br>γ-himachalene (3.13%)<br>cis-isoeugenol (1.99%)<br>linalool (1.79%)	*Listeria monocytogenes, E. coli, Staphylococcus aureus, A. section nigri* and *A. flavus*	0.05%	[11]
Lippia rugosa EO	*Lippia rugosa* leaves	geraniol (51.5%)<br>nerol (18.6%)	*A. flavus*	0.1%	[12]
Citrus reticulata EO	*Citrus reticulata* fruits	limonene (46.7%)<br>geranial (19.0%)<br>neral (14.5%)<br>geranyl acetate (3.9%)	*A. flavu*s	0.075%	[13]
Bay laurel EO	Bay laurel leaves	eucalyptol (27.2%)<br>α-terpinenyl acetate (10.2%)	*E. coli* and *A. flavus*	2.0%	[14]
Lemongrass EO	*Cymbopogon flexousus*	citral (76.00%)<br>neral(28%)	*Acinetobacter baumannii, Salmonella enteritidis*, *E. coli* and *A. flavus*	2.0%	[15]
Tea tree EO	Branches	cis-sabinine hydrate (38.43%)	*A. flavus*, *L. monocytogenes*, *Salmonella typhimurium* and *E. coli*	0.1%	[16]
Thyme EO	Thyme leaves	carvacrol (81%)<br>p-cymene (4.5%)	*Candida albicans* and *E. coli*	0.03%	[17]
Vetiver EO	Vetiver leaves	zizanoic acid (12.87%),<br>khusimol (11.48%)	*Staphylococcus aureus*	0.008%	[17]
Marigold EO	Marigold leaves	piperitone (50.7%)	*A. flavus*	0.2%	[18]
Spearmint EO	Spearmint leaves	carvone (51.7%),<br>cis-carveol (24.3%)	*A. flavus*	0.3%	[19]
Ocimum basilicum EO	Basil	linalool (56.7-60.6%),	*A. flavus*	0.3%	[20]

		epi-α-cadinol (8.6-11.4%)			
Caraway EO	Caraway seeds	carvone (44.5-95.9%)<br>limonene (1.5-51.3%)	*A. flavus*, *C. albicans* and *C. dubliniensis*	0.2%	[21]
Chamomile EO	Chamomile flower-heads	chamazulene (61.3%)<br>isopropyl hexadecanoate (12.7%)<br>trans-farnesol (6.9%)	*L. monocytogenes*, *Staphylococcus aureus*, *E. coli* and *Pseudomonas aeruginosa*	0.05%	[6]
Ageratum onyzoides EO	Ageratum conyzoides leaves	ageratocromene (34.69%)<br>caryophyllene (21.2%)	*A. flavus*, *Rhizoctonia solani*, *Sclerotium rolfsii*, *Botryodiplodia theobromae*, *Phomopsis theae* and *Fusarium* spp.	0.3%	[22]
Mandarin EO	Mandarin fruits	limonene (74.4%)<br>cis-oxide limonene (2.8%)	*Lactobacillus curvatus*, *Lactobacillus sakei*, *Staphylococcus carnosus*, *Staphylococcus xylosus*, *Enterobacter gergoviae* and *Enterobacter amnigenus*	0.94%	[23]
Grapefruit EO	Grapefruit peel	limonene (88.6%)<br>α-terpinene (1%)	*A. flavus*, *A. niger*, *Penicillium chrysogenum* and *Penicillium verrucosum*	0.94%	[24]
Orange EO	Orange fruits	limonene (85.5%)	*A. niger*, *A. flavus*, *Penicillium chrysogenum* and *Penicillium verrucosum*	0.94%	[24]
Zataria multiflora Boiss EO	*Zataria multiflora Boiss*	thymol (37.59%)<br>carvacrol (33.65%)<br>para-cymene (7.72%)	*Staphylococcus aureus*, *Bacillus cereus*, *E. coli*, *Salmonella typhi*, *Proteus vulgaris* and *Shigella flexneri*	0.04%	[25]
Piper betle L. EO	*Piper betle* leaves	eugenol (63.39%)<br>acetyleugenol (14.05%)	*A. flavus*	0.03-0.73%	[26]
Jamrosa EO	*Jamrosa* grass	(Z)-citral (59.69%)<br>linalyl acetate (34.99%)	*A. flavus*	0.04%	[27]
Cinnamomum zeylanicum Blume EO	*Lauraceae* barks	(E)-cinnamaldehyde (68.95%)<br>benzaldehyde (9.94%)	*Staphylococcus aureus*, *Streptococcus pyogenes*, *Streptococcus pneumoniae*,	0.04%-1.12%	[28]

		(E)-cinnamyl acetate (7.44%)	*Enterococus feacalis, Enterococcus faecium, Bacillus cereus, Acinetobacter lwoffii, Enterobacter aerogenes, E. coli, Proteus mirabilis, Pseudomonas aeruginosa, Salmonella typhimurium, L. monocytogenes, L. ivanovii, L. welshimeri, C. albicans, C. parapsilosis* and *C. krusei*

Table S3. Primers used for qRT-PCR

Species	Gene	Primer sequence (5' to 3')	Amplicon length (bp)
A. flavus	*β-tubulin*	F: CTCAATTCCGACCTGCGAAA	263
		R: ACGCCACGCATTTGATCTTC	
	aflR	F: GATCTGGCTGGTCAGGAGCA	204
		R: CGCCTGAAACGGTGGTAGTG	
	aflS	F: CTCGATGCGGCAGTGTATCT	109
		R: ACACCTCCACATGAGCCTTG	
	aflT	F: GATTCTATTGCCTTGATTTTGG	81
		R: GGCGTAGTGCCCTGTCTTAT	
	laeA	F: AAAGGTTGCTCGCTGGTACA	121
		R: GACTTCTGACGAAATGCGCC	
	brlA	F: TCTAGCGGGGATGACCTCAA	131
		R: CCGAAGGAAGCCAAAAGTGC	
A. ochraceus	*GADPH*	F: TGCTCAAGTACGACAGCACC	101
		R: CTCGGCGAAGAACTGAACCT	
	pks	F: TTCTCTGCGCTTCTCACATC	225
		R: AACATCATAAGAGGTCAACA	
	p450-B03	F: CTCGGTGACATCAGGGGTATC	470
		R: AGCGTATTCAGTCACTCATTCAGA	
	pacC	F: CTCATCGACCCTGCTCTGTC	121
		R: GACGCACCTTTTCAACCCAC	

References

1. Li, W.; Wang, P. J.; Shigematsu, M.; Lu, Z. G., Chemical Composition and Antimicrobial Activity of Essential Oil from *Amomum Tsao-Ko* Cultivated in Yunnan Area. *Advanced Materials Research* **2011,** 183-185, 910-914.
2. Diao, W. R.; Zhang, L. L.; Feng, S. S.; Xu, J. G., Chemical composition, antibacterial activity, and mechanism of action of the essential oil from *Amomum kravanh*. *Journal of Food Protection* **2014,** 77, (10), 1740.
3. Sindhu, S.; Chempakam, B.; Leela, N. K.; Suseela Bhai, R., Chemoprevention by essential oil of turmeric leaves (*Curcuma longa L.*) on the growth of *Aspergillus flavus* and aflatoxin production. *Food and Chemical Toxicology* **2011,** 49, (5), 1188-1192.
4. Stoilova, I.; Krastanov, A.; Stoyanova, A.; Denev, P.; Gargova, S., Antioxidant activity of a ginger extract (*Zingiber officinale*). *Food Chemistry* **2007,** 102, (3), 764-770.
5. Prakash, B.; Singh, P.; Kedia, A.; Singh, A.; Dubey, N. K., Efficacy Of Essential Oil Combination Of *Curcuma Longa L.* And *Zingiber Officinale Rosc.* As a Postharvest Fungitoxicant, Aflatoxin Inhibitor And Antioxidant Agent. *Journal of Food Safety* **2012,** 32, (3), 279-288.
6. Roby, M. H. H.; Sarhan, M. A.; Selim, K. A.-H.; Khalel, K. I., Antioxidant and antimicrobial activities of essential oil and extracts of fennel (*Foeniculum vulgare L.*) and chamomile (*Matricaria chamomilla L.*). *Industrial Crops and Products* **2013,** 44, 437-445.
7. Hajlaoui, H.; Trabelsi, N.; Noumi, E.; Snoussi, M.; Fallah, H.; Ksouri, R.; Bakhrouf, A., Biological activities of the essential oils and methanol extract of tow cultivated mint species (*Mentha longifolia* and *Mentha pulegium*) used in the Tunisian folkloric medicine. *World Journal of Microbiology and Biotechnology* **2009,** 25, (12), 2227-2238.
8. Sivropoulou, A.; Kokkini, S.; Lanaras, T.; Arsenakis, M., Antimicrobial activity of mint essential oils. *J.agric.food Chem* **1995,** 43, (9), 2384-2388.
9. Ejechi, B. O.; Nwafor, O. E.; Okoko, F. J., Growth inhibition of tomato-rot fungi by phenolic acids and essential oil extracts of pepperfruit (*Dennetia tripetala*). *Food Research International* **1999,** 32, (6), 395-399.
10. Pinto, E.; Vale-Silva, L.; Cavaleiro, C.; Salgueiro, L., Antifungal activity of the clove essential oil from *Syzygium aromaticum* on *Candida*, *Aspergillus* and dermatophyte species. *J Med Microbiol* **2009,** 58, (Pt 11), 1454-1462.
11. Elgayyar, M.; Draughon, F. A.; Golden, D. A.; Mount, J. R., Antimicrobial activity of essential oils from plants against selected pathogenic and saprophytic microorganisms. *Journal of Food Protection* **2001,** 64, (7), 1019-1024.
12. Tatsadjieu, N. L.; Dongmo, P. M. J.; Ngassoum, M. B.; Etoa, F. X.; Mbofung, C. M. F., Investigations on the essential oil of *Lippia rugosa* from Cameroon for its potential use as antifungal agent against *Aspergillus flavus* Link ex. Fries. *Food Control* **2009,** 20, (2), 161-166.
13. Singh, P.; Shukla, R.; Kumar, A.; Prakash, B.; Singh, S.; Dubey, N. K., Effect of *Citrus reticulata* and *Cymbopogon citratus* essential oils on *Aspergillus flavus* growth and aflatoxin production on *Asparagus racemosus*. *Mycopathologia* **2010,** 170, (3), 195-202.
14. Ramos, C.; Teixeira, B.; Batista, I.; Matos, O.; Serrano, C.; Neng, N. R.; Nogueira, J. M.; Nunes, M. L.; Marques, A., Antioxidant and antibacterial activity of essential oil and

extracts of bay laurel *Laurus nobilis Linnaeus* (Lauraceae) from Portugal. *Nat Prod Res* **2012,** 26, (6), 518-529.

15. Adukwu, E. C.; Bowles, M.; Edwards-Jones, V.; Bone, H., Antimicrobial activity, cytotoxicity and chemical analysis of lemongrass essential oil (*Cymbopogon flexuosus*) and pure citral. *Appl Microbiol Biotechnol* **2016,** 100, (22), 9619-9627.
16. Shellie, R.; Marriott, P.; Cornwell, C., Characterization and Comparison of Tea Tree and Lavender Oils by Using Comprehensive Gas Chromatography. *Journal of High Resolution Chromatography* **2000,** 23, (9), 554-560.
17. Hammer, K. A.; Carson, C. F.; Riley, T. V., Antimicrobial activity of essential oils and other plant extracts. *Journal of Applied Microbiology* **1999,** 86, (6), 985-990.
18. Soliman, K. M.; Badeaa, R. I., Effect of oil extracted from some medicinal plants on different mycotoxigenic fungi. *Food and Chemical Toxicology* **2002,** 40, (11), 1669-1675.
19. Hussain, A. I.; Anwar, F.; Shahid, M.; Ashraf, M.; Przybylski, R., Chemical Composition, and Antioxidant and Antimicrobial Activities of Essential Oil of Spearmint (*Mentha spicata L.*) From Pakistan. *Journal of Essential Oil Research* **2010,** 22, (1), 78-84.
20. Oxenham, S. K.; Svoboda, K. P.; Walters, D. R., Antifungal Activity of the Essential Oil of Basil (*Ocimum basilicum*). *Journal of Phytopathology* **2005,** 153, (3), 174–180.
21. Sahari, M. A.; Barzegar, M.; Darougheh, F., Antioxidant and Anti-fungal Effect of Caraway (*Carum Carvi L.*) Essential Oil in Real Food System. *Current Nutrition & Food Science* **2014,** 10, (1), 70-76.
22. Esper, R. H.; Gonçalez, E.; Felicio, R. C.; Felicio, J. D., Fungicidal activity and constituents of *Ageratum conyzoides* essential oil from three regions in São Paulo state, Brazil. *Arquivos do Instituto Biológico* **2015,** 82, (0), 1-4.
23. Viuda-Martos, M.; Ruiz-Navajas, Y.; Fernández-López, J.; Pérez-Álvarez, J., Antifungal activity of lemon (*Citrus lemon L.*), mandarin (*Citrus reticulata L.*), grapefruit (*Citrus paradisi L.*) and orange (*Citrus sinensis L.*) essential oils. *Food Control* **2008,** 19, (12), 1130-1138.
24. Mondello, L.; Dugo, P.; Bartle, K. D.; Dugo, G.; Cotroneo, A., Automated HPLC-HRGC: A powerful method for essential oils analysis. Part V. identification of terpene hydrocarbons of bergamot, lemon, mandarin, sweet orange, bitter orange, grapefruit, clementine and mexican lime oils by coupled HPLC-HRGC-MS(ITD). *Flavour and Fragrance Journal* **1995,** 10, (1), 33-42.
25. Sharififar, F.; Moshafi, M. H.; Mansouri, S. H.; Khodashenas, M.; Khoshnoodi, M., In vitro evaluation of antibacterial and antioxidant activities of the essential oil and methanol extract of endemic *Zataria multiflora Boiss*. *Food Control* **2007,** 18, (7), 800-805.
26. Prakash, B.; Shukla, R.; Singh, P.; Kumar, A.; Mishra, P. K.; Dubey, N. K., Efficacy of chemically characterized *Piper betle L.* essential oil against fungal and aflatoxin contamination of some edible commodities and its antioxidant activity. *International Journal of Food Microbiology* **2010,** 142, (1), 114-119.
27. Mishra, P. K.; Shukla, R.; Singh, P.; Prakash, B.; Kedia, A.; Dubey, N. K., Antifungal, anti-aflatoxigenic, and antioxidant efficacy of *Jamrosa* essential oil for preservation of herbal raw materials. *International Biodeterioration & Biodegradation* **2012,** 74, 11-16.
28. Unlu, M.; Ergene, E.; Unlu, G. V.; Zeytinoglu, H. S.; Vural, N., Composition,

antimicrobial activity and in vitro cytotoxicity of essential oil from *Cinnamomum zeylanicum* Blume (Lauraceae). *Food Chem Toxicol* **2010,** 48, (11), 3274-80.
